# Circulating miRNAs as liquid biopsy biomarkers for diagnosis in patients with colorectal cancer: a systematic review and meta-analysis

**DOI:** 10.3389/fgene.2025.1574586

**Published:** 2025-07-28

**Authors:** Sydney Schwab, Taichiro Nonaka

**Affiliations:** ^1^ School of Medicine, Louisiana State University Health Shreveport, Shreveport, LA, United States; ^2^ Department of Cellular Biology and Anatomy, Louisiana State University Health Sciences Center, Shreveport, LA, United States; ^3^ Feist-Weiller Cancer Center, Louisiana State University Health Shreveport, Shreveport, LA, United States

**Keywords:** colorectal cancer, biomarker, circulating biomarker, liquid biopsy, saliva diagnostics

## Abstract

**Objective:**

Colorectal cancer (CRC) remains a leading cause of cancer-related mortality worldwide, emphasizing the need for noninvasive and reliable diagnostic tools. Circulating microRNAs (miRNAs) have emerged as promising liquid biopsy biomarkers for CRC detection. This meta-analysis aimed to evaluate the diagnostic accuracy of blood- and saliva-derived miRNAs in CRC, assessing their sensitivity, specificity, and overall clinical potential.

**Methods:**

A comprehensive literature search was conducted across PubMed, Web of Science, and Scopus to identify relevant studies published between 2004 and 2024. Eligible studies included those that evaluated miRNA expression in plasma, serum, or saliva of CRC patients. A random-effects model was applied to calculate pooled sensitivity, specificity, diagnostic odds ratio (DOR), and area under the receiver operating characteristic curve (AUC). Heterogeneity was assessed using Cochrane’s Q test and I^2^ statistics, and risk of bias was evaluated using the QUADAS-2 tool.

**Results:**

A total of 37 studies with 2,775 patients were included in this meta-analysis. The pooled diagnostic performance demonstrated an AUC of 0.87 for combined blood- and saliva-derived miRNAs and 0.86 for blood-derived miRNAs alone, with both categories showing a sensitivity of 0.76 and specificity of 0.83. The diagnostic likelihood ratio (DLR) analysis yielded DLR positive values > 4 and DLR negative values < 0.3, indicating strong discriminatory ability. The DOR was 15.98 for combined blood- and saliva-derived miRNAs and 15.49 for blood-derived miRNAs alone, highlighting their comparable diagnostic potential. These findings suggest that circulating miRNAs serve as reliable biomarkers for CRC detection.

**Conclusion:**

This meta-analysis supports the diagnostic utility of circulating miRNAs as noninvasive biomarkers for CRC detection, with saliva-derived miRNAs offering a potential complementary role. Blood-based miRNA analysis demonstrated high diagnostic accuracy, and the integration of saliva-derived miRNAs slightly improved AUC. Future research should focus on standardizing miRNA panels and validation in larger, independent cohorts to facilitate their clinical application in CRC screening and early detection.

## 1 Introduction

Colorectal cancer (CRC) is one of the most prevalent malignancies worldwide and a leading cause of cancer-related mortality, particularly in developed countries (Xi and Xu, 2021). According to global cancer statistics, CRC ranks third in terms of incidence and second in cancer-related deaths (Sung et al., 2021). The incidence of CRC varies geographically, with higher rates observed in Western nations compared to developing regions, a pattern linked to lifestyle factors such as diet, obesity, and physical inactivity (Akimoto et al., 2021). Epidemiological studies indicate that aging is a significant risk factor, with the majority of cases diagnosed in individuals over the age of 50 (Siegel et al., 2020). However, in recent years, there has been a concerning rise in early-onset CRC cases among younger adults, highlighting the need for revised screening guidelines and increased awareness of early detection strategies (Sinicrope, 2022).

The etiology of CRC is complex and multifactorial, involving both genetic and environmental factors (Kuipers et al., 2015). Sporadic CRC, which accounts for approximately 70% of cases, develops due to the accumulation of somatic mutations and epigenetic alterations (Angelakas et al., 2024). Hereditary forms, including Lynch syndrome and familial adenomatous polyposis (FAP), contribute to about 5%–10% of cases and are associated with a higher risk of early-onset CRC (Jasperson et al., 2010). At the molecular level, CRC follows distinct pathways of tumorigenesis, including chromosomal instability (CIN), microsatellite instability (MSI), and CpG island methylator phenotype (CIMP), each characterized by unique genetic and epigenetic alterations (Boland and Goel, 2010; Pino and Chung, 2010; Toyota et al., 1999). These mechanisms drive key oncogenic events, such as mutations in the *APC*, *KRAS*, *TP53*, and *BRAF* genes, which contribute to the initiation and progression of CRC (Nguyen and Duong, 2018).

Despite advances in treatment, CRC continues to have a high mortality rate, particularly in patients diagnosed at advanced stages (Koroukian et al., 2023). The 5-year survival rate for localized CRC exceeds 90%, but this figure drops significantly to below 15% for patients with metastatic disease (Siegel et al., 2020). The primary reason for this contrast in survival rates is the late detection of CRC in a large proportion of patients. Early-stage CRC is often asymptomatic, and by the time symptoms such as rectal bleeding, changes in bowel habits, or abdominal pain appear, the disease has frequently progressed to an advanced stage (Mitchell E. et al., 2008). This indicates the urgent need for improved screening and early detection strategies to enhance patient outcomes and reduce CRC-related mortality.

Current CRC screening methods include colonoscopy, stool-based tests, and imaging modalities such as computed tomography (CT) colonography (Shaukat and Levin, 2022). While colonoscopy remains the gold standard for CRC detection due to its high sensitivity and ability to remove precancerous polyps, it is invasive, costly, and associated with patient discomfort (Andronis et al., 2024). As a result, compliance with colonoscopy screening remains an issue. Stool-based tests, such as the fecal occult blood test (FOBT) and fecal immunochemical test (FIT), offer noninvasive alternatives but have limited sensitivity, particularly for detecting early-stage CRC and precancerous lesions (Gomez-Molina et al., 2024). These limitations indicate the need for a more effective, noninvasive, and widely accessible screening approach that can improve early detection rates and patient compliance.

To address these challenges, liquid biopsy has emerged as a promising alternative for CRC detection and monitoring (Mauri et al., 2022). Liquid biopsy involves the analysis of circulating biomarkers, which provide real-time insights into tumor dynamics. The three primary types of circulating biomarkers include circulating tumor DNA (ctDNA), circulating tumor cells (CTCs), and circulating RNA molecules (Ma et al., 2024). Each of these biomarkers offers unique advantages and limitations in reflecting the characteristics of CRC tumors.

Among these, ctDNA is considered one of the most promising biomarkers due to its ability to capture tumor-specific genetic mutations such as *KRAS*, *BRAF*, and *TP53* (Nakamura et al., 2024). Highly sensitive techniques such as PCR and next-generation sequencing (NGS) enable the detection of even minute amounts of ctDNA in the bloodstream, making it a valuable tool for early diagnosis and treatment monitoring. However, ctDNA primarily provides information on genetic alterations and does not reflect other critical aspects of tumor biology, such as cellular interactions or phenotypic heterogeneity.

In contrast, CTCs provide a more comprehensive picture of tumor biology, as they contain complete cellular structures, including RNA and proteins (Lin et al., 2021). This allows for a broader molecular analysis, including assessments of metastatic potential and treatment resistance. However, due to their rarity in circulation, detecting CTCs is technically challenging, and they may not always be representative of the entire tumor (Castro-Giner and Aceto, 2020). Because tumors are often heterogeneous, CTCs might capture only a limited subset of tumor cells, reducing their overall clinical applicability in CRC screening.

Circulating RNA molecules, particularly microRNAs (miRNAs), have gained increasing attention due to their stability in blood and their role in regulating gene expression in cancer (Schwarzenbach et al., 2014). In circulation, miRNAs exist in different forms: they can be freely circulating, protein-bound, or encapsulated within extracellular vesicles such as exosomes (Metcalf, 2024). Exosomes are small vesicles secreted by tumor cells and are believed to carry biomolecules reflective of the entire tumor, making them particularly valuable for studying tumor heterogeneity (Ghosh et al., 2024). Unlike ctDNA, which represents specific genomic alterations within particular signaling pathways, and CTCs, which may capture only certain tumor subpopulations, circulating miRNAs have the potential to comprehensively reflect gene regulation and signaling pathways across virtually all tumor cells, providing a more holistic representation of tumor biology (Tan et al., 2021; Li et al., 2022). This broader molecular representation makes them an attractive biomarker source for CRC detection and monitoring.

Recent studies have demonstrated that circulating miRNAs can serve as early diagnostic markers for CRC, with specific miRNA expression profiles distinguishing CRC patients from healthy individuals (Table 1). Their dynamic nature allows for real-time assessment of tumor progression and treatment response, positioning circulating miRNAs as essential tools in precision oncology. While blood-based liquid biopsy is currently the most explored approach, saliva-based liquid biopsy is gaining interest as a completely noninvasive diagnostic method with the potential for frequent at-home monitoring (Nonaka and Wong, 2022).

**TABLE 1:** Characteristics of the studies included in the meta-analysis.

Study ID, author, year	miRNA	No. of patients	TP	FP	FN	TN	Sample	Method	Ref
Ng et al. (2009a)	miR-17-3p	90	58	15	32	35	Plasma	RT-PCR	Ng et al. (2009)
Ng et al. (2009b)	miR-92	90	80	15	10	35	Plasma	RT-PCR	Ng et al. (2009)
Huang et al. (2010a)	miR-92a	100	65	11	35	48	Plasma	qRT-PCR	Huang et al. (2010)
Huang et al. (2010b)	miR-29a	100	69	6	31	53	Serum	qRT-PCR	Huang et al. (2010)
Pu et al. (2010)	miR-221	103	89	22	14	15	Plasma	qRT-PCR	Pu et al. (2010)
Kannan et al. (2012)	miR-21	20	18	2	2	18	Plasma	RT-PCR	Kanaan et al. (2012)
Wang et al. (2017)	miR-21	32	28	10	4	29	Serum	qRT-PCR	Wang and Zhang (2012)
Feng et al. (2013)	miR-29a	98	74	6	24	44	Serum	qRT-PCR	Feng et al. (2013)
Giraldez et al. (2013)	miR-29a	21	13	3	8	17	Serum	qRT-PCR	Giraldez et al. (2013)
Liu et al. (2013a)	miR-92a	200	131	14	69	66	Serum	qRT-PCR	Liu et al. (2013)
Liu et al. (2013b)	miR-21	200	130	12	70	68	Serum	qRT-PCR	Liu et al. (2013)
Luo et al. (2013)	miR-16	80	42	27	38	117	Plasma	qRT-PCR	Luo et al. (2013)
Song et al. (2013)	miR-21	40	30	17	10	39	Serum	qRT-PCR	Song et al. (2013)
Toiyama et al. (2013)	miR-21	186	154	5	32	48	Serum	qRT-PCR	Toiyama et al. (2013)
Brunet Vega et al. (2013)	miR-29a	30	23	1	7	25	Serum	qRT-PCR	Brunet Vega et al. (2013)
Nonaka et al. (2014)	miR-21	84	46	5	38	27	Serum	qRT-PCR	Nonaka et al. (2014)
Ogata-Kawata et al. (2014)	miR-451	88	54	1	34	10	Serum	qRT-PCR	Ogata-Kawata et al. (2014)
Zanutto et al. (2014)	miR-378	29	20	6	9	23	Plasma	qRT-PCR	Zanutto et al. (2014)
Zhang et al. (2014)	miR-106a	50	31	15	19	32	Plasma	qRT-PCR	Zhang et al. (2014)
Chen et al. (2015a)	miR-20a	100	46	21	54	58	Plasma	qRT-PCR	Chen et al. (2015)
Chen et al. (2015b)	miR-106a	100	74	44	26	35	Plasma	qRT-PCR	Chen et al. (2015)
Lv et al. (2015)	miR-155	146	85	3	61	57	Serum	qRT-PCR	Lv et al. (2015)
Zhu et al. (2015)	miR-17-3p	70	59	19	11	51	Serum	qRT-PCR	Zhu et al. (2015)
Bastaminejad et al. (2017)	miR-21	40	34	11	6	29	Serum	qRT-PCR	Bastaminejad et al. (2017)
Krawczyk et al. (2017a)	miR4316	54	33	11	21	59	Plasma	qRT-PCR	Krawczyk et al. (2017)
Krawczyk et al. (2017b)	miR-506	54	41	28	13	42	Plasma	qRT-PCR	Krawczyk et al. (2017)
Sazanov et al. (2017a)	miR-21	31	20	5	11	29	Plasma	qRT-PCR	Sazanov et al. (2017)
Shi et al. (2017)	miR-155	90	62	32	28	58	Serum	qRT-PCR	Shi et al. (2017)
Wang et al. (2017)	miR-210	268	205	27	63	75	Serum	qRT-PCR	Wang et al. (2017)
Xu and Gu (2017)	miR-196b	103	90	37	13	63	Serum	qRT-PCR	Xu and Gu (2017)
Zhu et al. (2017)	miR-183	46	30	17	16	29	Serum	qRT-PCR	Zhu et al. (2017)
Bilegsaikhan et al. (2018)	miR-338-5p	80	53	9	27	71	Serum	qRT-PCR	Bilegsaikhan et al. (2018)
Liu et al. (2018)	miR-21	85	71	0	14	78	Serum	qRT-PCR	Liu et al. (2018)
Tayel et al. (2018)	miRNA-200c	30	26	8	4	22	Serum	qRT-PCR	Tayel et al. (2018)
Sabry et al. (2019a)	miRNA-21	35	32	5	3	96	Serum	qRT-PCR	Sabry et al. (2019)
Sabry et al. (2019b)	miRNA-210	35	31	10	4	91	Serum	qRT-PCR	Sabry et al. (2019)
Bader El Din et al. (2020)	miR-21	60	48	0	12	30	Serum	qRT-PCR	Bader El Din et al. (2020)
Elaguizy et al. (2020)	miR-21	50	42	5	8	45	Serum	qRT-PCR	Elaguizy et al. (2020)
Farouk et al. (2020)	miR-21	35	29	1	5	34	Serum	qRT-PCR	Farouk et al. (2020)
Ghareib et al. (2020)	miR-21	48	46	4	2	44	Serum	qRT-PCR	Ghareib et al. (2020)
Li et al. (2020)	miR-21	40	36	3	4	17	Serum	qRT-PCR	Li et al. (2020)
Nassar et al. (2021)	miR-21	62	24	6	38	38	Plasma	qRT-PCR	Nassar et al. (2021)
Sazanov et al. (2017b)	miR-21	34	33	3	1	31	Saliva	qRT-PCR	Sazanov et al. (2017)
Rapado-Gonzalez et al. (2019)	miRNA panel*	51	37	6	14	13	Saliva	qRT-PCR	Rapado-Gonzalez et al. (2019)

The two saliva-based substudies Sazanov et al. (2017b) and Rapado-Gonzalez et al. (2019) are placed at the bottom of the table for clarity. *miRNA panel includes miR-186-5p, miR-29a-3p, miR29c-3P, miR766-3p, and miR-491-5p. TP, true positive; FP, false positive; FN, false negative; TN, true negative; qRT-PCR, quantitative reverse transcription PCR.

Saliva-based liquid biopsy offers several advantages over traditional blood-based methods (Nonaka and Wong, 2023). Studies indicate that 20%–30% of the salivary proteome overlaps with the plasma proteome, highlighting the close biological relationship between saliva and blood (Yan et al., 2009; Bandhakavi et al., 2009; Denny et al., 2008). Tumor-derived extracellular vesicles can enter circulation and reach salivary glands, where they are taken up by acinar cells through endocytosis or membrane fusion (Nonaka and Wong, 2017). This suggests that salivary miRNAs may serve as robust CRC biomarkers, enabling noninvasive screening with minimal patient discomfort. Unlike venipuncture, saliva collection is simple, painless, and can be performed frequently, facilitating continuous disease monitoring and early detection. The integration of blood and salivary miRNA analyses may further enhance diagnostic accuracy by capturing a broader spectrum of tumor-derived biomarkers, leading to improved sensitivity and specificity in CRC detection (Nguyen and Nonaka, 2024).

Given the potential of circulating miRNAs as liquid biopsy biomarkers, this meta-analysis aims to evaluate their diagnostic utility in CRC detection. Additionally, we explore the benefits of integrating blood and salivary miRNA data to determine whether a combined approach can improve diagnostic accuracy and provide a more comprehensive molecular panel for CRC screening. By investigating these aspects, we seek to contribute to the development of a minimally invasive, highly accurate, and widely accessible screening method for colorectal cancer, ultimately improving early detection rates and patient outcomes.

## 2 Materials and methods

### 2.1 Search strategy

**FIGURE 1:**
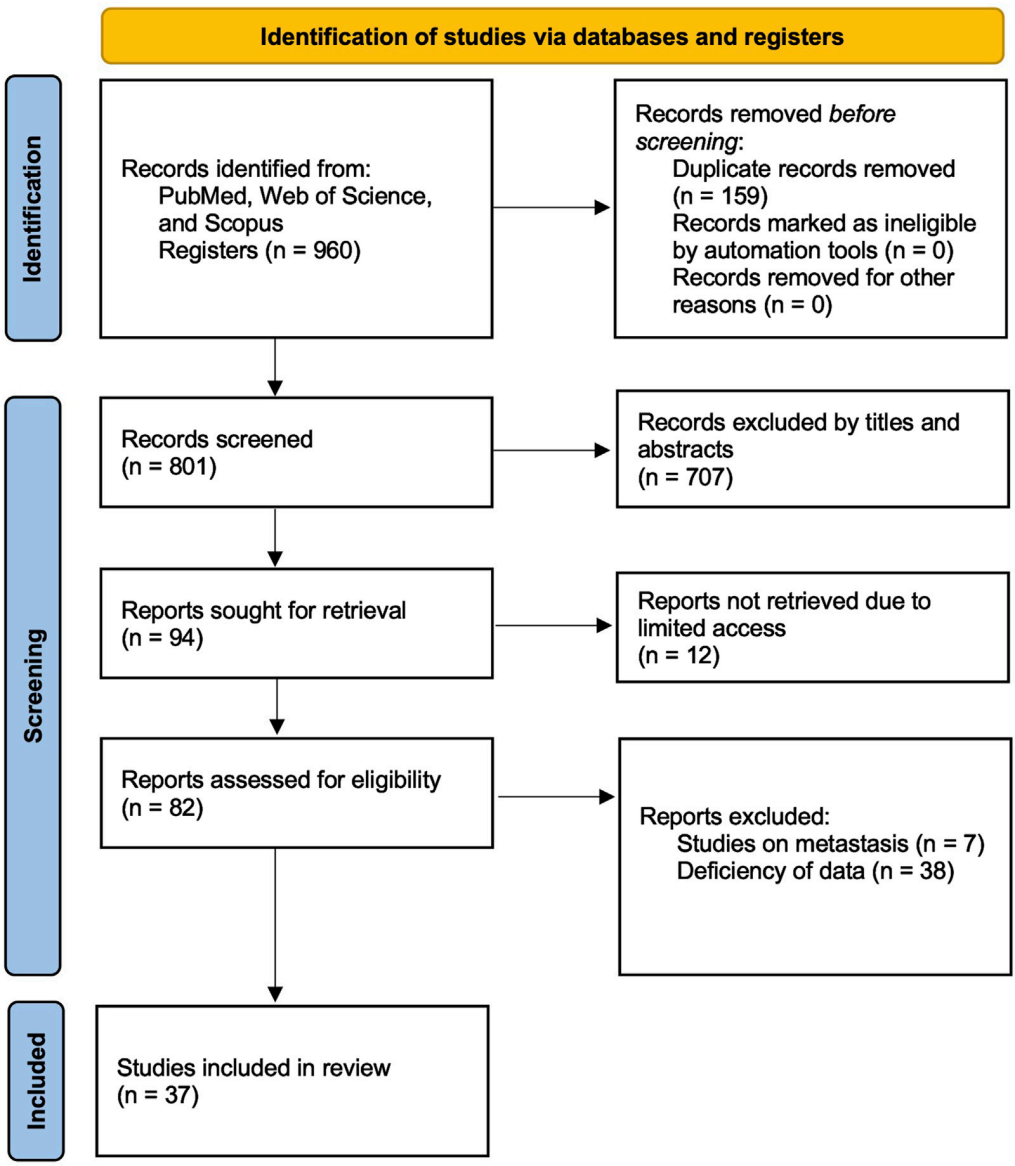
PRISMA flow diagram illustrating the study selection process. The diagram illustrates the number of records identified, screened, excluded, and included in the systematic review, in accordance with the Preferred Reporting Items for Systematic Reviews and Meta-Analyses (PRISMA) guidelines.

To ensure a comprehensive and up-to-date meta-analysis on circulating miRNAs as liquid biopsy biomarkers for colorectal cancer (CRC), a systematic literature search was conducted across PubMed, Web of Science, and Scopus. The search was structured to identify studies that evaluated the diagnostic performance of circulating miRNAs in CRC patients. A combination of relevant keywords and Medical Subject Headings (MeSH) terms related to colorectal cancer, microRNA (or miRNA), liquid biopsy, and diagnosis was employed. Specifically, the following search string was applied: (“colorectal cancer” OR “colon cancer” OR “rectal cancer” OR “colorectal neoplasm*” OR “colon neoplasm*” OR “rectal neoplasm*“) AND (“microRNA*” OR “miRNA*” OR “circulating microRNA*” OR “circulating miRNA*”) AND (“blood” OR “plasma” OR “serum” OR “saliva”) AND (“diagnos*” OR “detect*” OR “screen*” OR “liquid biopsy”). The search specifically targeted studies that utilized biofluid specimens, including plasma, serum, and saliva, while excluding those that primarily analyzed urine or other less commonly used fluids. Boolean operators (AND, OR) were strategically applied to refine search results and maximize relevant study retrieval. The search was restricted to peer-reviewed articles published between 2004 and 2024 to ensure relevance to current methodologies and advancements in miRNA detection. Studies were eligible for inclusion if they were indexed in major databases and had English-language abstracts available, provided that sufficient data for meta-analysis could be extracted. Additionally, the reference lists of included studies were screened to identify any additional relevant publications. The study selection process, including the number of articles screened at each stage, is illustrated in the Preferred Reporting Items for Systematic Reviews and Meta-Analyses (PRISMA) flow diagram (Figure 1). This systematic review and meta-analysis was conducted in accordance with the PRISMA 2020 guidelines, and a completed PRISMA 2020 checklist is provided in Supplementary Table S1.

### 2.2 Eligibility criteria

Strict inclusion and exclusion criteria were implemented to ensure that only relevant studies were included in the meta-analysis. Studies were eligible if they assessed the diagnostic accuracy of circulating miRNAs in CRC patients using plasma, serum, or saliva as the specimen type. They were required to provide sufficient data for constructing a 2 × 2 contingency table, including true positive (TP), false positive (FP), true negative (TN), and false negative (FN). To maintain statistical validity, only studies with at least 10 CRC patients and 10 healthy control subjects were included. This decision was made to exclude extremely small studies, which could produce unstable estimates, while balancing inclusiveness and comprehensiveness. This threshold was pragmatically determined, in line with common practice in diagnostic meta-analyses. Furthermore, studies had to use validated detection methodologies such as PCR, microarrays, or NGS. Studies were excluded if they focused on non-human subjects, were case reports, reviews, editorials, or conference abstracts that lacked original data. Studies that did not investigate human plasma, serum, or saliva-derived miRNAs or failed to provide adequate data for statistical analysis were also excluded. In cases where multiple studies reported overlapping or redundant data, the most comprehensive and recent study was selected for inclusion.

### 2.3 Data extraction and quality assessment

Following the eligibility screening, full-text articles that met the inclusion criteria underwent systematic data extraction. Extracted data were categorized into three key areas: study characteristics, miRNA features, and diagnostic performance metrics. Study characteristics included the first author’s name, year of publication, study location, number of CRC patients, and number of healthy controls. Information on miRNA features included the specific miRNAs analyzed, detection methods used, and type of biological specimen examined. Diagnostic performance metrics extracted from each study included TP, FP, TN, and FN values necessary for subsequent meta-analysis calculations.

To assess methodological quality, the Quality Assessment of Diagnostic Accuracy Studies-2 (QUADAS-2) tool was employed (Whiting et al., 2011). This tool evaluates four primary domains: patient selection, index test, reference standard, and flow and timing. Each domain was assessed for risk of bias and applicability concerns. The risk of bias for each study was categorized as low, high, or unclear based on QUADAS-2 evaluations. RevMan (v.5.4) software was used to generate a graphical summary of the quality assessment across all included studies.

### 2.4 Statistical analysis

All statistical analyses were performed using Stata (v.18) and RevMan (v.5.4). A random-effects model was applied to account for variability among studies and estimate pooled diagnostic accuracy metrics. The primary statistical measures included pooled sensitivity, specificity, diagnostic likelihood ratios (DLR positive and negative), diagnostic score (DS), and diagnostic odds ratio (DOR). Hierarchical summary receiver operating characteristic (HSROC) curves were plotted, and the area under the curve (AUC) was calculated to evaluate the overall diagnostic performance of circulating miRNAs in CRC detection. Heterogeneity among studies was assessed using Cochran’s Q test and the I^2^ statistic. A statistically significant Q test (p < 0.05) or an I^2^ value exceeding 50% was considered indicative of substantial heterogeneity. Publication bias was assessed using Deeks’ funnel plot asymmetry test, where a p-value <0.05 was considered indicative of significant bias. All statistical tests were two-sided, and a p-value <0.05 was deemed statistically significant. The use of rigorous statistical methodologies aimed to provide reliable and clinically meaningful insights into the diagnostic utility of circulating miRNAs for CRC detection. By integrating a robust search strategy, well-defined eligibility criteria, meticulous data extraction, quality assessment, and advanced statistical analyses, this meta-analysis sought to provide comprehensive and high-quality evidence on the role of circulating miRNAs as noninvasive biomarkers for CRC diagnosis.

### 2.5 Visualization of study and diagnostic characteristics

To visually summarize the distribution of miRNAs across studies and their corresponding diagnostic performances, a Sankey plot was generated using Python (version 3.10) with the Plotly library (Plotly version 5.18.0). For each miRNA included in the meta-analysis, sensitivity and specificity were calculated based on the extracted true positive (TP), false positive (FP), false negative (FN), and true negative (TN) values. These calculated metrics were then categorized into “High (≥0.85)”, “Moderate (0.70-0.84)”, or “Low (<0.70)” groups based on the predefined thresholds. The Sankey plot was constructed using these classifications to depict the flow from individual miRNAs to their specificity and sensitivity levels. This visualization aimed to highlight the most frequently studied miRNAs and provide a comprehensive overview of their diagnostic accuracy profiles.

## 3 Results

### 3.1 Study selection

A systematic literature search was conducted across PubMed, Web of Science, and Scopus, identifying 960 records. After removing 159 duplicates, 801 studies remained for screening. Based on title and abstract evaluation, 707 studies were excluded as they did not meet the inclusion criteria. The full texts of 94 studies were retrieved for in-depth assessment, but 12 studies were inaccessible, and 45 studies were excluded due to insufficient data or relevance. Ultimately, 37 studies that evaluated the diagnostic accuracy of blood- and saliva-derived miRNAs in CRC patients were included in this meta-analysis. The study selection process is summarized in the PRISMA flow diagram (Figure 1). Several papers contained multiple substudies, each investigating different miRNAs separately. These were considered as independent studies in the meta-analysis. As a result, the total number of included substudies increased to 44, with 42 focusing on blood-derived miRNAs and two focusing on saliva-derived miRNAs. In cases where multiple substudies were derived from the same publication, they were distinguished by adding letters after the publication year [e.g., Ng et al. (2009a), Ng et al. (2009b)] (Table 1).

### 3.2 Study characteristics

Among the 44 included substudies, 42 investigated miRNAs in plasma or serum, while two evaluated miRNAs in saliva [i.e., Sazanov et al. (2017b) and Rapado-Gonzalez et al. (2019)]. One of these studies exclusively focused on saliva-derived miRNAs, while the other assessed both saliva- and blood-derived miRNAs. To maintain clarity in data presentation, the two studies involving saliva miRNAs are placed at the bottom of all tables and forest plots. The included studies varied in publication year, sample size, specific miRNAs analyzed, detection methodology, and sample type. Most studies employed quantitative reverse transcription PCR (qRT-PCR) for miRNA detection, while a smaller subset used conventional RT-PCR. Sample sizes ranged from 10 to over 200 CRC patients, with control groups consisting of healthy individuals. Most studies investigated single miRNA biomarkers, some analyzed two miRNAs, and one study examined a miRNA panel to improve diagnostic accuracy. A detailed summary of study characteristics is provided in Table 1.

### 3.3 Quality assessment

**FIGURE 2:**
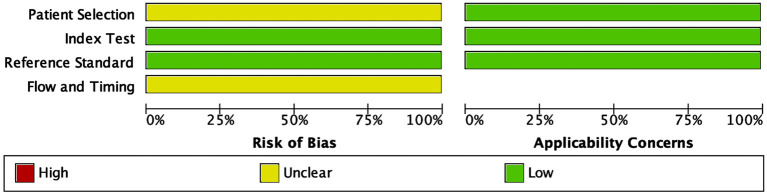
Quality assessment of the included studies using the Quality Assessment of Diagnostic Accuracy Studies 2 (QUADAS-2) tool. The figure displays the proportions of included studies rated as low, unclear, or high risk of bias across four domains: patient selection, index test, reference standard, and flow and timing. Applicability concerns are shown separately for the first three domains. Green indicates low risk/concern, yellow indicates unclear, and red indicates high.

The methodological quality of the included studies was evaluated using the Quality Assessment of Diagnostic Accuracy Studies-2 (QUADAS-2) tool. This assessment covered four key domains: patient selection, index test, reference standard, and flow and timing. The risk of bias and applicability concerns were categorized as low, unclear, or high, with the results summarized in Figure 2. In the patient selection domain, most studies did not explicitly report whether participants were enrolled consecutively or randomly. Due to insufficient reporting, this domain was classified as having an unclear risk of bias (yellow). For the index test domain, many studies did not specify whether the miRNA assays were performed with blinding to the reference standard, leading to an unclear risk of bias classification. No major concerns were raised regarding their applicability as miRNA detection methods were appropriate for CRC diagnostics.

The reference standard domain exhibited a low risk of bias, as all included studies utilized histopathological confirmation as the gold standard for CRC diagnosis. This consistency enhances the reliability of the diagnostic accuracy measures. The flow and timing domain showed an unclear risk of bias in some studies due to insufficient reporting on whether all patients underwent the same reference standard within a uniform timeframe. All applicability concerns were low, indicating that the included studies were appropriate for evaluating the diagnostic performance of circulating miRNAs in CRC detection. The QUADAS-2 assessment confirmed that the included studies were of acceptable quality for meta-analysis, supporting the robustness and clinical relevance of the findings.

### 3.4 Meta-analysis

**FIGURE 3:**
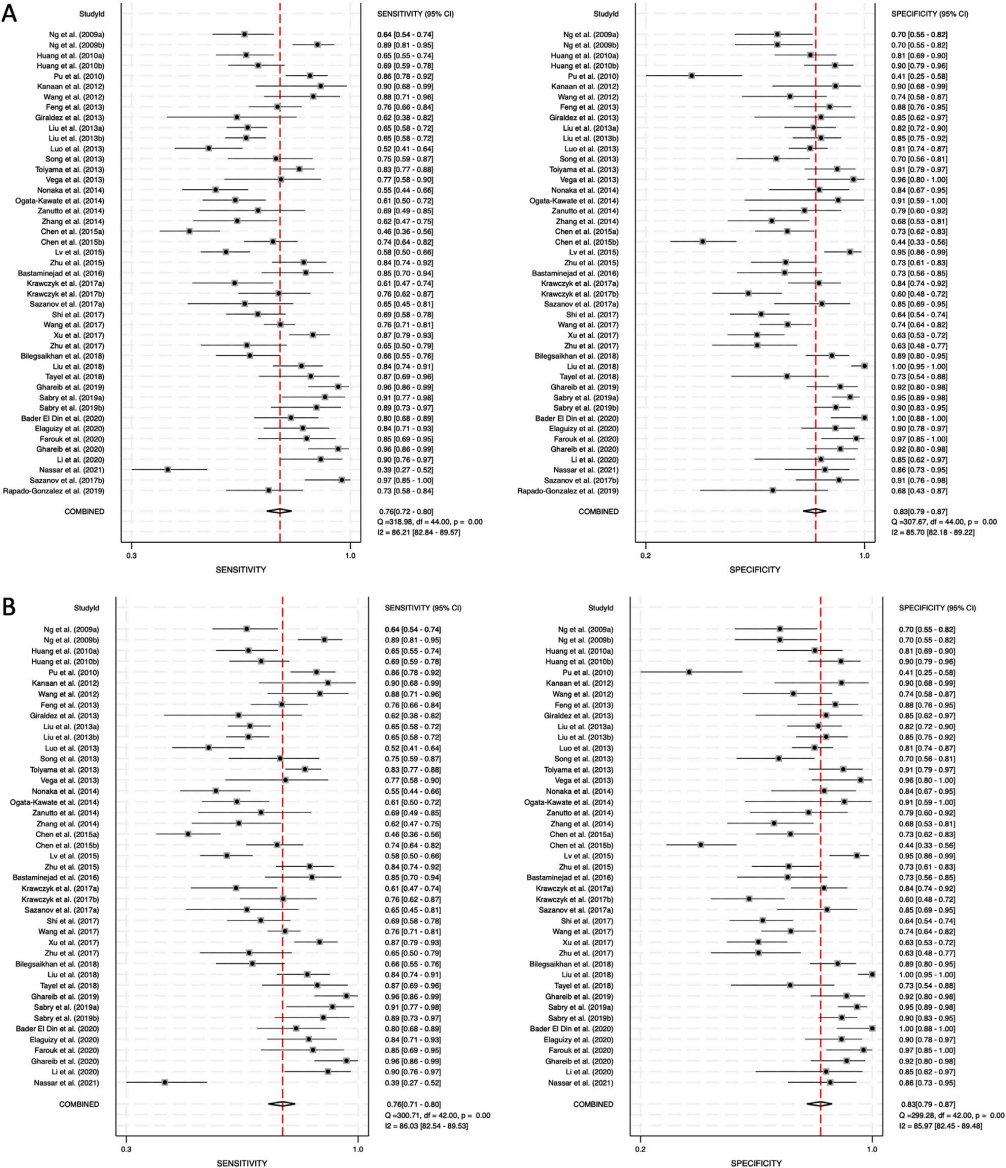
Pooled sensitivity and specificity for circulating miRNA-based diagnostics of colorectal cancer. **(A)** Combined blood- and saliva-derived miRNAs (Sensitivity: 0.76, Specificity: 0.83). **(B)** Blood-derived miRNAs alone (Sensitivity: 0.76, Specificity: 0.83).

The diagnostic performance of circulating miRNAs was analyzed in two categories: combined blood- and saliva-derived miRNAs, followed by a separate analysis of blood-derived miRNAs alone. Due to the limited number of studies focusing exclusively on saliva-derived miRNAs, a separate meta-analysis for this category was not performed. Heterogeneity was assessed using Cochrane’s Q test and I^2^ statistics, revealing substantial variation among studies. For combined blood- and saliva-derived miRNAs, the I^2^ value for sensitivity was 86.21% (95% CI: 82.84–89.57), and for specificity, it was 85.70% (95% CI: 82.18–89.22), indicating considerable heterogeneity (Figure 3A). Similarly, for blood-derived miRNAs alone, the I^2^ value for sensitivity was 86.03% (95% CI: 82.54–89.53), and for specificity, it was 85.97% (95% CI: 82.45–89.48) (Figure 3B). Given these findings, a random-effects model was applied to account for variability across studies.

**FIGURE 4:**
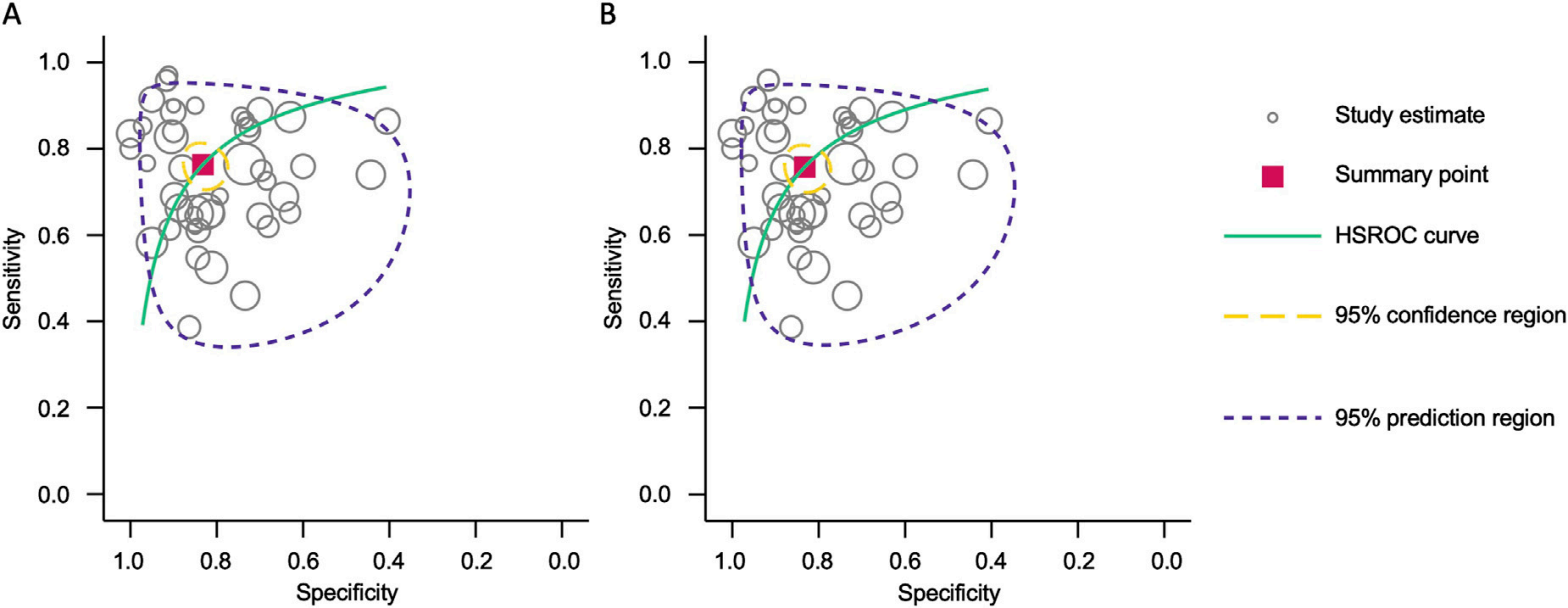
Hierarchical summary receiver operating characteristic (HSROC) curve analysis for circulating miRNA-based colorectal cancer diagnostics. **(A)** Combined blood- and saliva-derived miRNAs (AUC: 0.87, 95% CI: 0.83–0.89). **(B)** Blood-derived miRNAs alone (AUC: 0.86, 95% CI: 0.83–0.89).

Forest plots displaying sensitivity and specificity, along with HSROC curves, were generated for both categories. The combined blood- and saliva-derived miRNAs had a sensitivity of 0.76 (95% CI: 0.72–0.80) and a specificity of 0.83 (95% CI: 0.79–0.87), with an AUC of 0.87 (95% CI: 0.83–0.89), indicating strong diagnostic accuracy (Figures 3A, 4A). For blood-derived miRNAs alone, the sensitivity was 0.76 (95% CI: 0.71–0.80), specificity was 0.83 (95% CI: 0.79–0.87), and AUC was 0.86 (95% CI: 0.83–0.89) (Figures 3B, 4B). These findings indicate that circulating miRNAs provide a high level of diagnostic accuracy for CRC detection. The AUC values above 0.85 in both categories suggest that these biomarkers perform well in distinguishing CRC patients from healthy individuals. The sensitivity of 0.76 suggests that miRNAs correctly identify a high proportion of CRC cases, while the specificity of 0.83 indicates that they effectively exclude non-CRC cases. The strong performance of blood-derived miRNAs alone highlights their reliability as a noninvasive diagnostic tool for CRC. The inclusion of saliva-derived miRNAs resulted in a slight improvement in AUC, suggesting that adding saliva samples to liquid biopsy approaches may further enhance diagnostic accuracy.

**FIGURE 5:**
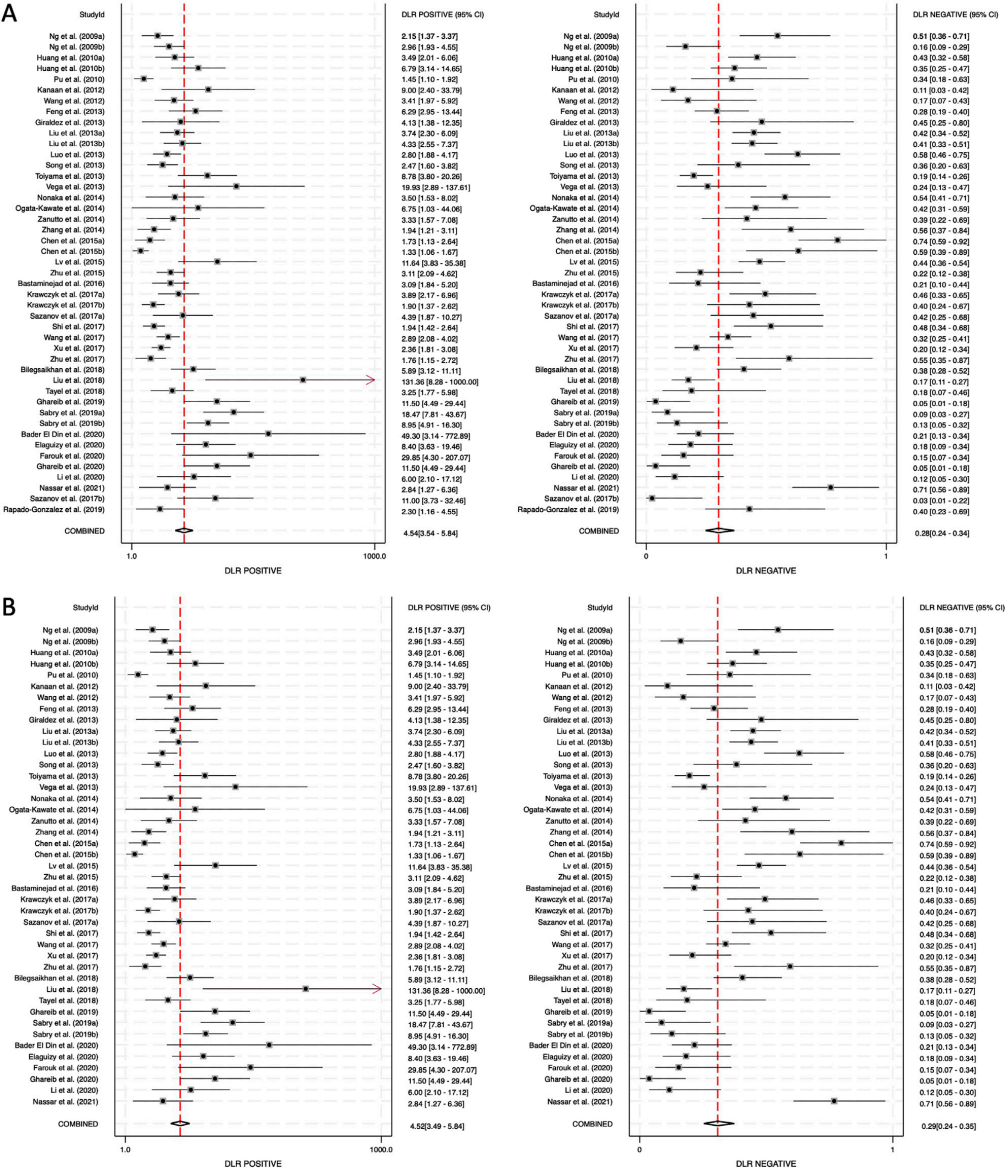
Diagnostic likelihood ratios (DLRs) for circulating miRNA-based colorectal cancer diagnostics. **(A)** Combined blood- and saliva-derived miRNAs (DLR positive: 4.54, DLR negative: 0.28). **(B)** Blood-derived miRNAs alone (DLR positive: 4.52, DLR negative: 0.29).

The positive diagnostic likelihood ratio (DLR positive) and negative diagnostic likelihood ratio (DLR negative) were also analyzed. The DLR positive for combined blood- and saliva-derived miRNAs was 4.54 (95% CI: 3.54–5.84), and the DLR negative was 0.28 (95% CI: 0.24–0.34) (Figure 5A). For blood-derived miRNAs alone, the DLR positive was 4.52 (95% CI: 3.49–5.84), and the DLR negative was 0.29 (95% CI: 0.24–0.35) (Figure 5B). A DLR positive greater than 4 suggests that the presence of these miRNAs significantly increases the likelihood of CRC diagnosis, indicating strong diagnostic value. Similarly, a DLR negative below 0.3 suggests that the absence of these miRNAs substantially reduces the likelihood of CRC, further reinforcing their potential utility as biomarkers. These values indicate that circulating miRNAs, both from blood and saliva, can serve as reliable diagnostic markers with strong discriminatory ability.

**FIGURE 6:**
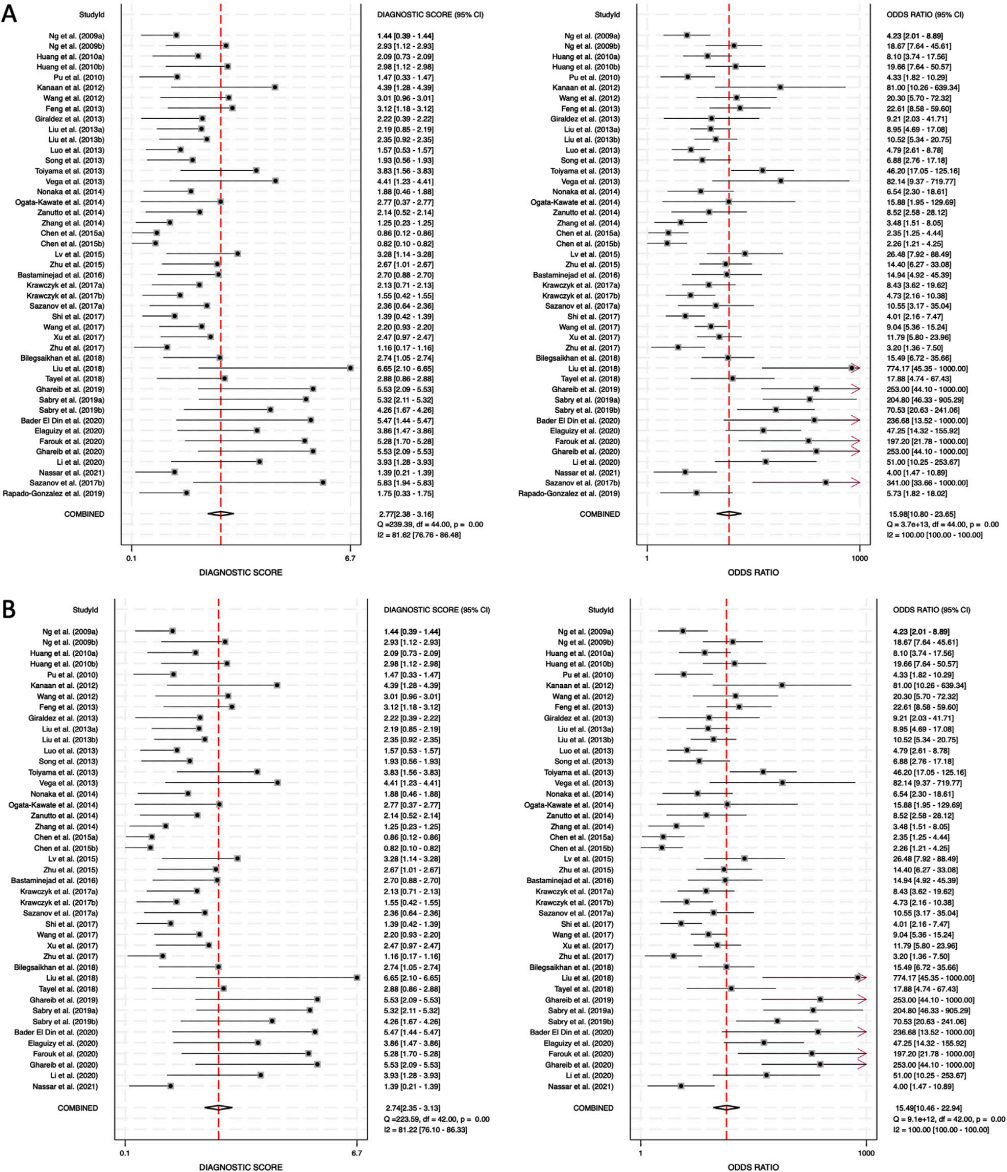
Diagnostic score (DS) and diagnostic odds ratio (DOR) for circulating miRNA-based colorectal cancer diagnostics. **(A)** Combined blood- and saliva-derived miRNAs (DS: 2.77, DOR: 15.98). **(B)** Blood-derived miRNAs alone (DS: 2.74, DOR: 15.49).

The DS and DOR were calculated to evaluate the overall diagnostic performance. For combined blood- and saliva-derived miRNAs, the DS was 2.77 (95% CI: 2.38–3.16), and the DOR was 15.98 (95% CI: 10.80–23.65) (Figure 6A). For blood-derived miRNAs alone, the DS was 2.74 (95% CI: 2.35–3.13), and the DOR was 15.49 (95% CI: 10.46–23.94) (Figure 6B). A higher DS indicates a greater ability of the miRNA biomarkers to differentiate CRC patients from controls. The similar diagnostic scores for both categories suggest that combined blood- and saliva-derived miRNAs provide nearly the same level of diagnostic performance as blood-derived miRNAs alone. Additionally, the DOR values greater than 10 confirm the strong overall discriminative power of these biomarkers, reinforcing their potential clinical utility for CRC detection. These results indicate that circulating miRNAs can serve as reliable, noninvasive diagnostic markers with high diagnostic accuracy.

In addition to the overall analysis, subgroup meta-analyses were conducted specifically for miR-21 and miR-29a, which were the most frequently evaluated individual miRNAs among the included studies. For miR-21, the pooled sensitivity was 0.83 (95% CI: 0.76–0.89), the specificity was 0.90 (95% CI: 0.85–0.93), and the AUC was 0.93 (95% CI: 0.91–0.95), indicating excellent diagnostic accuracy (Supplementary Figures S1, S3). For miR-29a, the pooled sensitivity was 0.72 (95% CI: 0.66–0.77), the specificity was 0.90 (95% CI: 0.84–0.94), and the AUC was 0.89 (95% CI: 0.86–0.92), demonstrating strong diagnostic performance as well (Supplementary Figures S2, S4). These findings suggest that miR-21, in particular, may serve as a highly accurate biomarker for CRC detection, and that miR-29a also holds promise as a reliable diagnostic candidate. The results of these subgroup analyses reinforce the diagnostic potential of specific miRNAs and help to address the heterogeneity observed when pooling a broader range of different miRNAs.

### 3.5 Publication bias

**FIGURE 7:**
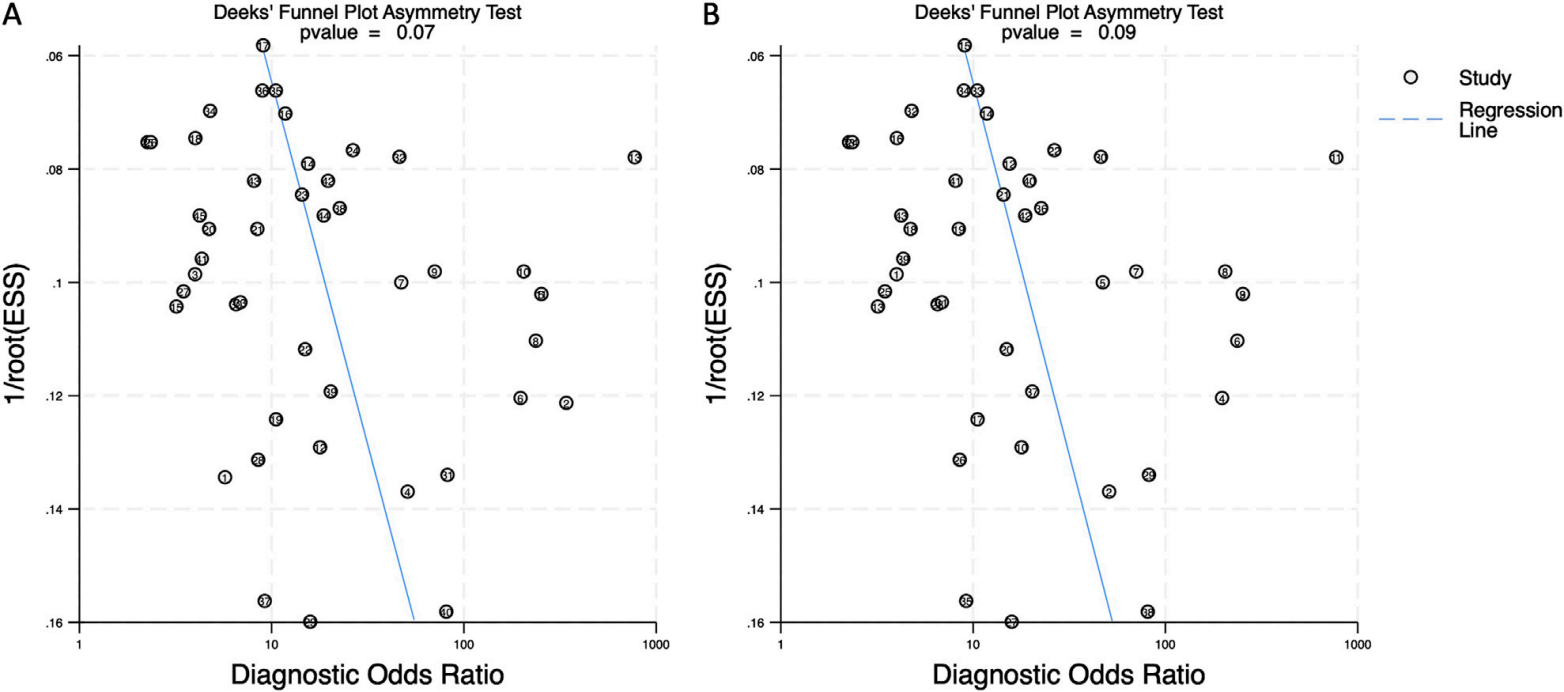
Deeks’ funnel plot assessing publication bias in circulating miRNA-based colorectal cancer diagnostics. **(A)** Combined blood- and saliva-derived miRNAs (p = 0.07). **(B)** Blood-derived miRNAs alone (p = 0.09).

Deeks’ funnel plots were generated to assess publication bias for both biomarker categories. The asymmetry test produced a p-value of 0.07 for combined blood- and saliva-derived miRNAs and 0.09 for blood-only miRNAs, indicating no significant publication bias in this meta-analysis (Figure 7).

### 3.6 Overview of miRNA distribution and diagnostic performance

**FIGURE 8:**
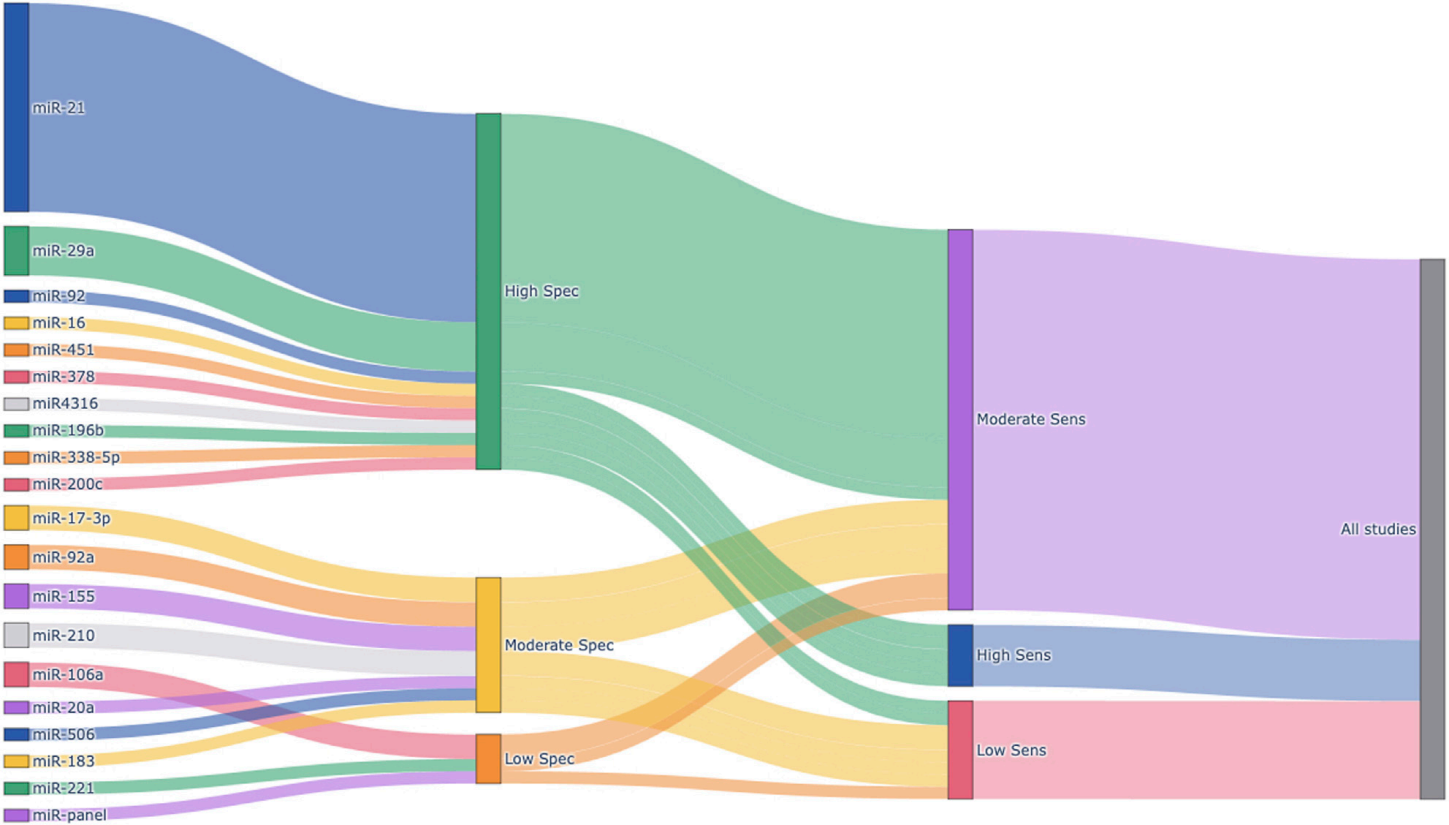
Sankey plot visualizing the distribution of circulating miRNAs and their diagnostic performance. Each node represents an individual miRNA (left), categorized specificity (middle), or categorized sensitivity (right). Sensitivity and specificity were calculated for each miRNA based on extracted TP, FP, FN, and TN values from the included studies. Diagnostic performance was classified into three categories: High (≥0.85), Moderate (0.70–0.84), and Low (<0.70). The width of the flows between nodes is proportional to the number of studies reporting each miRNA and its associated diagnostic category. Spec, Specificity; Sens, Sensitivity.

Figure 8 presents a Sankey plot summarizing the distribution of the miRNAs included in the meta-analysis and their corresponding specificity and sensitivity classifications. miR-21 was the most frequently studied miRNA, followed by miR-29a, miR-92a, and others. Most studies on miR-21 demonstrated high specificity and high-to-moderate sensitivity, indicating its strong diagnostic potential. Similarly, miR-29a exhibited high specificity and high-to-moderate sensitivity across included studies. Other miRNAs showed more variable diagnostic performances. This visual representation provides an intuitive understanding of the diagnostic potential and research trends of circulating miRNAs in colorectal cancer detection.

## 4 Discussion

This meta-analysis evaluated the diagnostic performance of circulating miRNAs in colorectal cancer (CRC) detection, comparing combined blood- and saliva-derived miRNAs with blood-derived miRNAs alone. The results demonstrated strong diagnostic accuracy for circulating miRNAs, with an AUC of 0.87 for combined blood- and saliva-derived miRNAs and 0.86 for blood-derived miRNAs alone, suggesting that both categories effectively distinguish CRC patients from healthy individuals. Sensitivity and specificity values were 0.76 and 0.83, respectively, for both groups. The inclusion of saliva-derived miRNAs resulted in a slight improvement in AUC, indicating a potential role for saliva-based liquid biopsy in enhancing CRC diagnosis. Diagnostic likelihood ratios further reinforced the clinical relevance of circulating miRNAs, with DLR positive values exceeding 4 and DLR negative values below 0.3, highlighting their strong discriminatory power. The DS and DOR analyses revealed that combined blood- and saliva-derived miRNAs (DOR = 15.98) and blood-derived miRNAs alone (DOR = 15.49) provided comparable diagnostic capabilities. These findings demonstrate the potential of circulating miRNAs as reliable, noninvasive biomarkers for CRC detection and suggest that the integration of saliva-based assays may offer an additional diagnostic advantage.

Subgroup analyses further revealed that specific miRNAs, particularly miR-21 and miR-29a, possess strong individual diagnostic performance. miR-21 demonstrated a sensitivity of 0.83, a specificity of 0.90, and an AUC of 0.93, while miR-29a showed a sensitivity of 0.72, a specificity of 0.90, and an AUC of 0.89. These findings reinforce the clinical utility of miR-21 as a highly accurate biomarker for CRC detection, and highlight the potential role of miR-29a as a promising diagnostic candidate.

In addition to the conventional meta-analytic findings, we incorporated a Sankey plot to visually illustrate the distribution of miRNAs and their diagnostic performance. This novel visualization method allowed us to concisely display which miRNAs were most frequently evaluated and how they were categorized in terms of specificity and sensitivity. It also emphasized the strong diagnostic potential of miR-21 and miR-29a, as well as the heterogeneity among other miRNAs. Such an approach enhances the interpretability of the systematic review and may guide future research efforts by highlighting promising biomarkers and research gaps.

miRNAs are small, non-coding RNA molecules that play a crucial role in post-transcriptional gene regulation (Bushati and Cohen, 2007). Their biogenesis follows a well-defined pathway, beginning with transcription into primary miRNAs (pri-miRNAs), processing into precursor miRNAs (pre-miRNAs), and subsequent cleavage by the Dicer enzyme to form mature miRNAs (Treiber et al., 2019). These mature miRNAs are incorporated into the RNA-induced silencing complex (RISC), where they regulate target mRNAs through degradation or translational repression (Krol et al., 2010). In normal physiological conditions, miRNAs regulate a wide range of cellular functions, including differentiation, proliferation, apoptosis, and immune responses (Mehta and Baltimore, 2016). However, in cancer cells, miRNA expression is often dysregulated, contributing to tumor progression, metastasis, and resistance to therapy (Lin and Gregory, 2015). Some miRNAs function as tumor suppressors by inhibiting oncogene expression, while others act as oncogenic miRNAs (oncomiRs) by suppressing tumor suppressor genes (Esquela-Kerscher and Slack, 2006). The balance between these miRNAs is critical in maintaining normal cellular homeostasis, and alterations in their expression profiles are a hallmark of cancer.

miRNAs are released into the extracellular environment through various mechanisms, including passive leakage from apoptotic or necrotic cells and active secretion via exosomes and other extracellular vesicles (Valadi et al., 2007). In the bloodstream, circulating miRNAs exist in three major forms: free-floating (naked) miRNAs, miRNAs bound to proteins such as Argonaute2 (AGO2) or high-density lipoproteins (HDL), and miRNAs encapsulated within exosomes (Arroyo et al., 2011; Vickers et al., 2011). The stability of these miRNAs varies, with naked miRNAs being highly susceptible to degradation by RNases, protein-bound miRNAs offering moderate protection, and exosomal miRNAs being the most stable due to their encapsulation within a lipid bilayer (Mitchell PS. et al., 2008). Among these, exosomal miRNAs represent the most promising biomarker candidates for liquid biopsy (Mori et al., 2019). Unlike naked and protein-bound miRNAs, which are often released from apoptotic or necrotic cells, exosomal miRNAs are actively secreted through a regulated pathway involving intracellular multivesicular bodies (MVBs) that fuse with the plasma membrane (Turchinovich et al., 2011; Gibbings et al., 2009). This mechanism ensures that exosomal miRNAs reflect the pathophysiological state of actively proliferating cancer cells, making them a highly relevant marker for real-time tumor monitoring.

When comparing ctDNA, CTCs, and exosomal miRNAs, their pathophysiological origins provide insights into their diagnostic potential. ctDNA is primarily derived from apoptotic and necrotic tumor cells, and its detection allows for highly sensitive cancer identification (Jahr et al., 2001). However, ctDNA analysis is limited to specific genetic alterations such as *KRAS* and *TP53* mutations, providing only partial information about tumor biology. CTCs, on the other hand, represent intact cancer cells circulating in the bloodstream. Their detection is useful for analyzing surface markers, such as PD-L1, which are critical for immunotherapy selection (Strati et al., 2017; Janning et al., 2019). However, the rarity of CTCs in circulation and their potential to only reflect a subpopulation of heterogeneous tumor cells limit their clinical applicability. In contrast, exosomal miRNAs are secreted by virtually all cancer cells, making them a more comprehensive representation of the tumor landscape (Nail et al., 2023). Since miRNAs regulate various oncogenic pathways, the miRNA profile in exosomes can provide extensive insights into the tumor’s molecular characteristics. While current techniques primarily analyze bulk exosomal miRNA populations, advancements in single-exosome analysis may enable more precise tumor characterization in the future (Ferguson et al., 2022).

In terms of diagnostic performance, ctDNA currently exhibits the highest sensitivity and specificity among liquid biopsy targets for CRC, with both metrics often exceeding 90% when detected using advanced technologies. Nevertheless, ctDNA testing primarily captures information on genetic mutations and requires complex, high-cost sequencing platforms. CTC analysis offers the unique advantage of cellular-level information, including protein expression relevant for therapeutic decisions, however, CTC assays generally suffer from low sensitivity due to the rarity of CTCs, particularly in early-stage disease. In contrast, circulating miRNAs provide a complementary approach. Although they may show slightly lower analytical performance compared to ctDNA, miRNAs offer practical advantages such as ease of collection, high stability in circulation, broader tumor representation beyond genetic mutations, and the ability to reflect dynamic tumor and stromal interactions. Moreover, miRNAs can be detected using simpler, more cost-effective techniques such as quantitative PCR. Comparative studies have suggested that miRNAs, especially when combined with other liquid biopsy components, may enhance early CRC detection and facilitate more comprehensive molecular profiling.

Colorectal cancer remains difficult to detect in early stages due to the absence of specific symptoms and the reluctance of patients to undergo colonoscopy (Kerrison et al., 2022; Adelstein et al., 2011). This reluctance contributes to delayed diagnoses and a higher proportion of cases being detected at advanced stages (Esteva et al., 2007). Therefore, there is a pressing need for more accessible and convenient screening methods that can encourage patient participation. While blood-based liquid biopsy presents a promising alternative for early CRC detection, saliva-based diagnostics offer an even more convenient and noninvasive approach. Saliva collection can be performed at home without specialized equipment, eliminating the need for venipuncture and increasing patient compliance in routine screening programs. Further advancements in the study of salivary miRNAs are expected to drive significant progress in this field, potentially revolutionizing colorectal cancer screening and offering a more accessible, patient-friendly approach to early detection.

Despite demonstrating the strong diagnostic potential of circulating miRNAs for CRC detection, this meta-analysis has several limitations. One major limitation is the significant heterogeneity observed across the included studies, as indicated by high I^2^ values for sensitivity (86.21% and 86.03%) and specificity (85.70% and 85.97%). This variability may stem from differences in study design, patient populations, sample collection methods, and miRNA detection techniques. All included studies employed targeted detection methods such as qRT-PCR or RT-PCR, which focus on predefined sets of miRNAs. The lack of exploratory technologies such as NGS or microarray platforms may introduce selection bias, as novel or less-studied miRNAs could have been underrepresented. Although a random-effects model was employed to mitigate this heterogeneity, the inconsistencies may impact the generalizability of the findings. Future research employing broader screening approaches would be valuable for identifying additional biomarker candidates and reducing potential biases associated with targeted analyses. Additionally, the number of studies exclusively investigating saliva-based miRNAs was limited. Consequently, a separate meta-analysis for saliva-derived miRNAs alone could not be conducted. Given the potential of saliva as a noninvasive liquid biopsy medium, further research is needed to validate the diagnostic performance of salivary miRNAs in larger, independent cohorts. Another important limitation is the lack of a universally accepted miRNA panel for CRC detection. The included studies examined different miRNA biomarkers, with some focusing on individual miRNAs and others on multi-miRNA panels. The absence of a standardized miRNA signature complicates direct comparisons and hinders clinical application. Future research should aim to establish a consensus on the most diagnostically relevant miRNA biomarkers to facilitate clinical implementation.

## 5 Conclusion

This meta-analysis provides valuable insights into the diagnostic potential of circulating miRNAs for CRC detection. The findings indicate the strong diagnostic performance of combined blood- and saliva-derived miRNAs, offering benefits for noninvasive cancer screening. However, addressing current limitations through larger multicenter studies, standardized methodologies, and prospective validation will be essential for advancing miRNA-based liquid biopsy toward routine clinical application. By refining diagnostic strategies and leveraging technological advancements, circulating miRNAs will become an essential component in CRC detection and precision medicine.

## Data Availability

The original contributions presented in the study are included in the article/Supplementary Material, further inquiries can be directed to the corresponding author.
