# Supplementary material for: Circulating miRNAs as liquid biopsy biomarkers for diagnosis in patients with colorectal cancer: a systematic review and meta-analysis
Source: Front Genet. 2025 Jul 28;16:1574586. doi: 10.3389/fgene.2025.1574586 (PMC12336027; doi:10.3389/fgene.2025.1574586)
Supplement: Supplementary file 1 [file Supplementaryfile1.pdf]

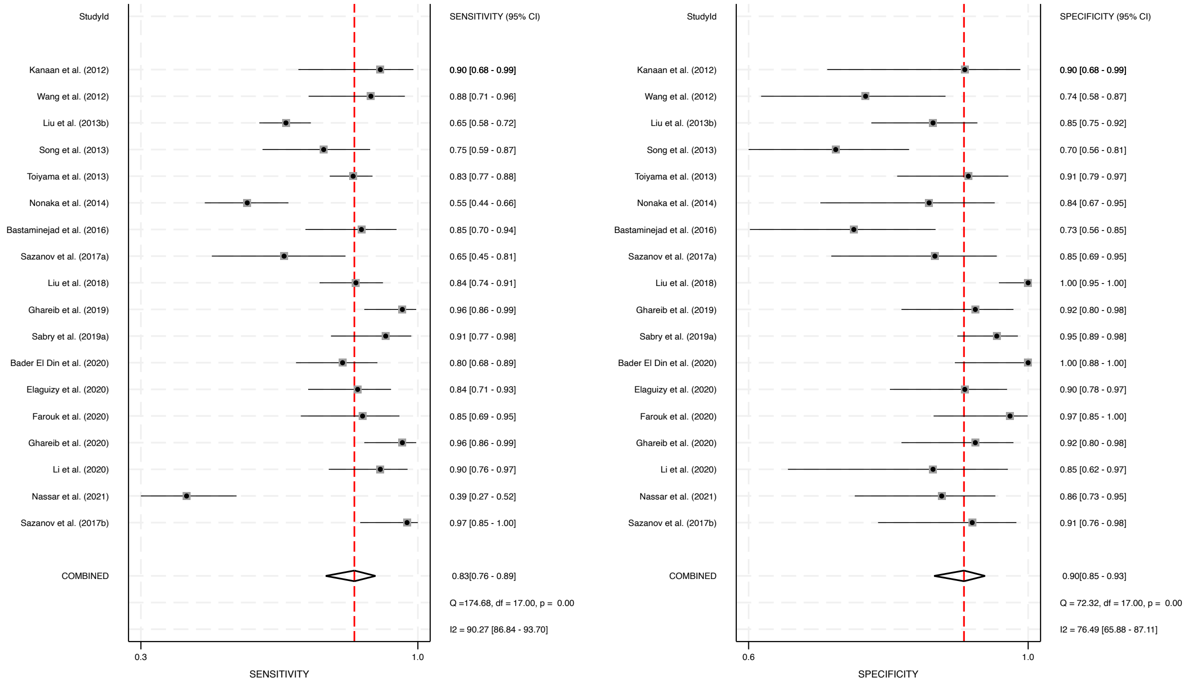

Supplementary Figure S1. Forest plot of pooled sensitivity and specificity for miR-21 in the diagnosis of colorectal cancer. This subgroup meta-analysis evaluated the diagnostic performance of miR-21 across the included studies. The pooled sensitivity was 0.83 (95% CI: 0.76-0.89), and the pooled specificity was 0.90 (95% CI: 0.85-0.93), indicating excellent diagnostic accuracy.

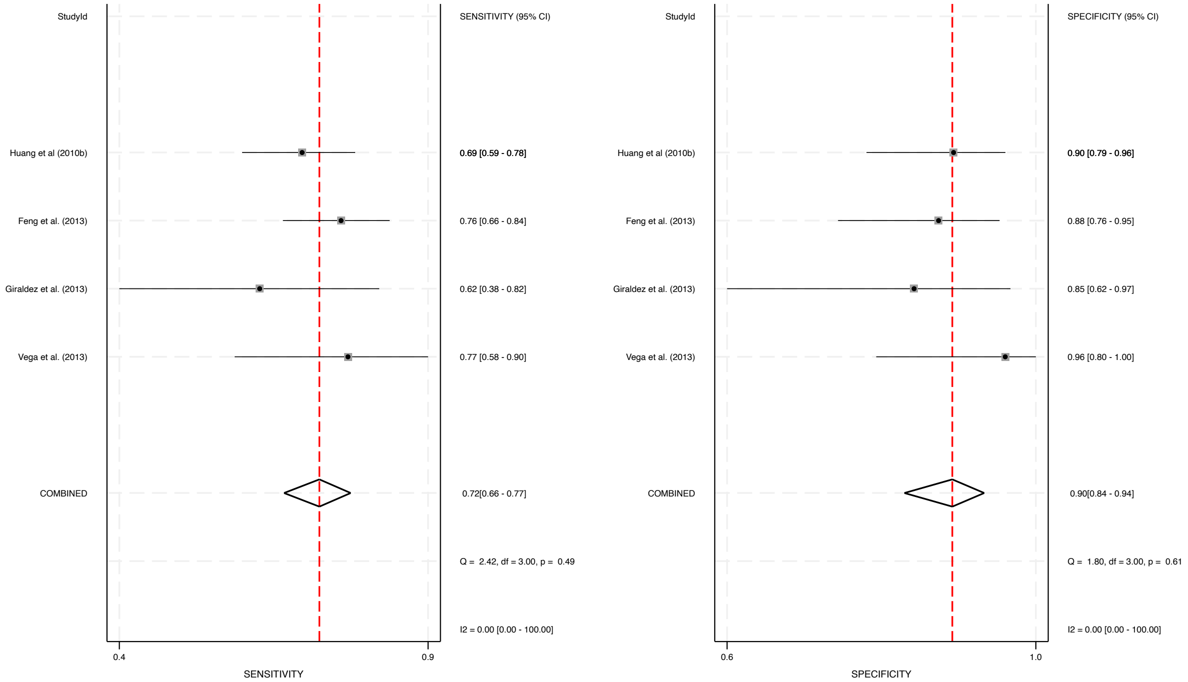

Supplementary Figure S2. Forest plot of pooled sensitivity and specificity for miR-29a in the diagnosis of colorectal cancer. This subgroup meta-analysis assessed the diagnostic performance of miR-29a. The pooled sensitivity was 0.72 (95% CI: 0.66-0.77), and the pooled specificity was 0.90 (95% CI: 0.84-0.94), demonstrating strong diagnostic accuracy.

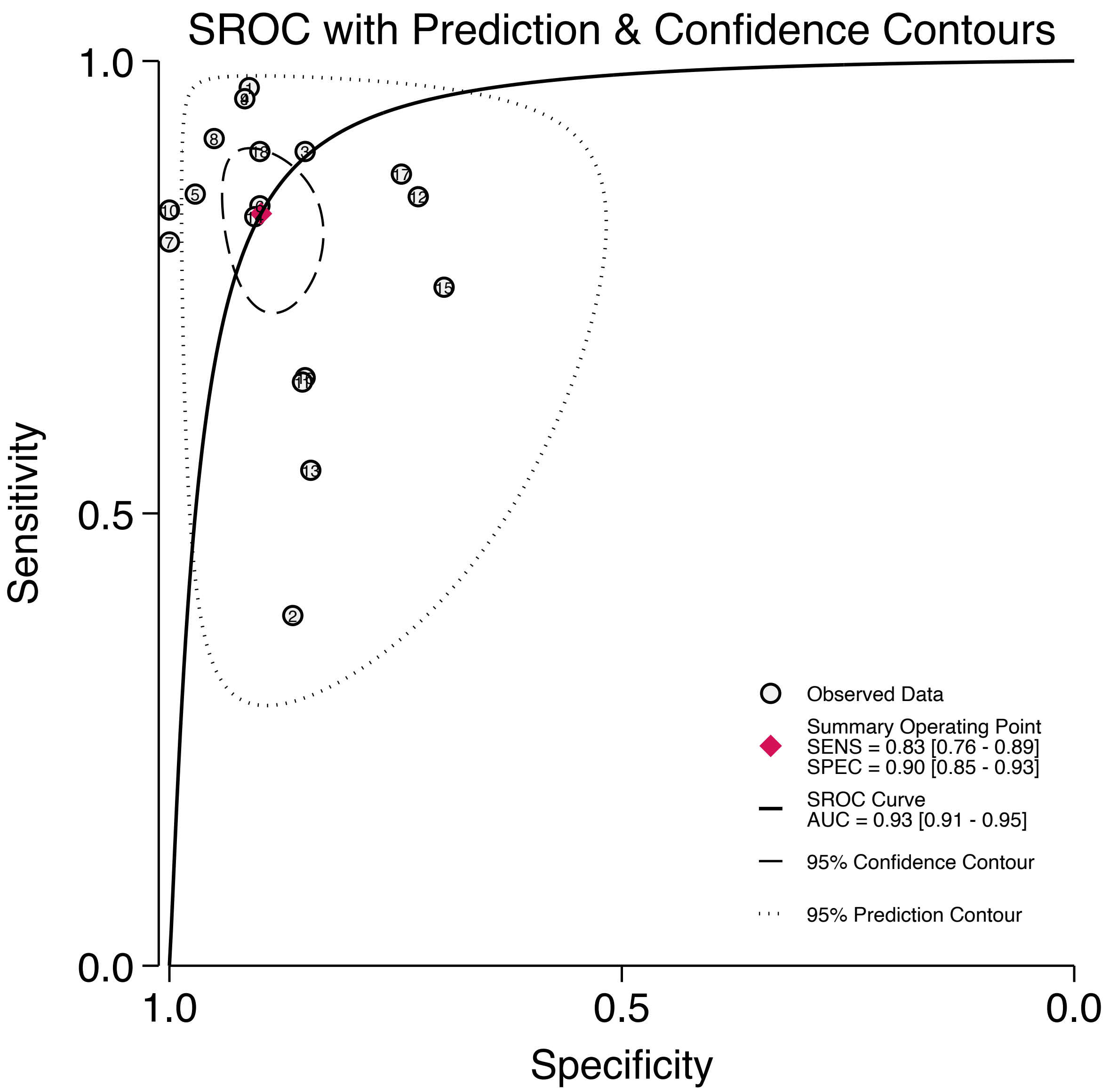

Supplementary Figure S3. Summary receiver operating characteristic (SROC) curve for miR-21. The SROC curve summarizes the diagnostic performance of miR-21 for colorectal cancer detection, with an area under the curve (AUC) of 0.93 (95% CI: 0.91-0.95), reflecting excellent overall accuracy.

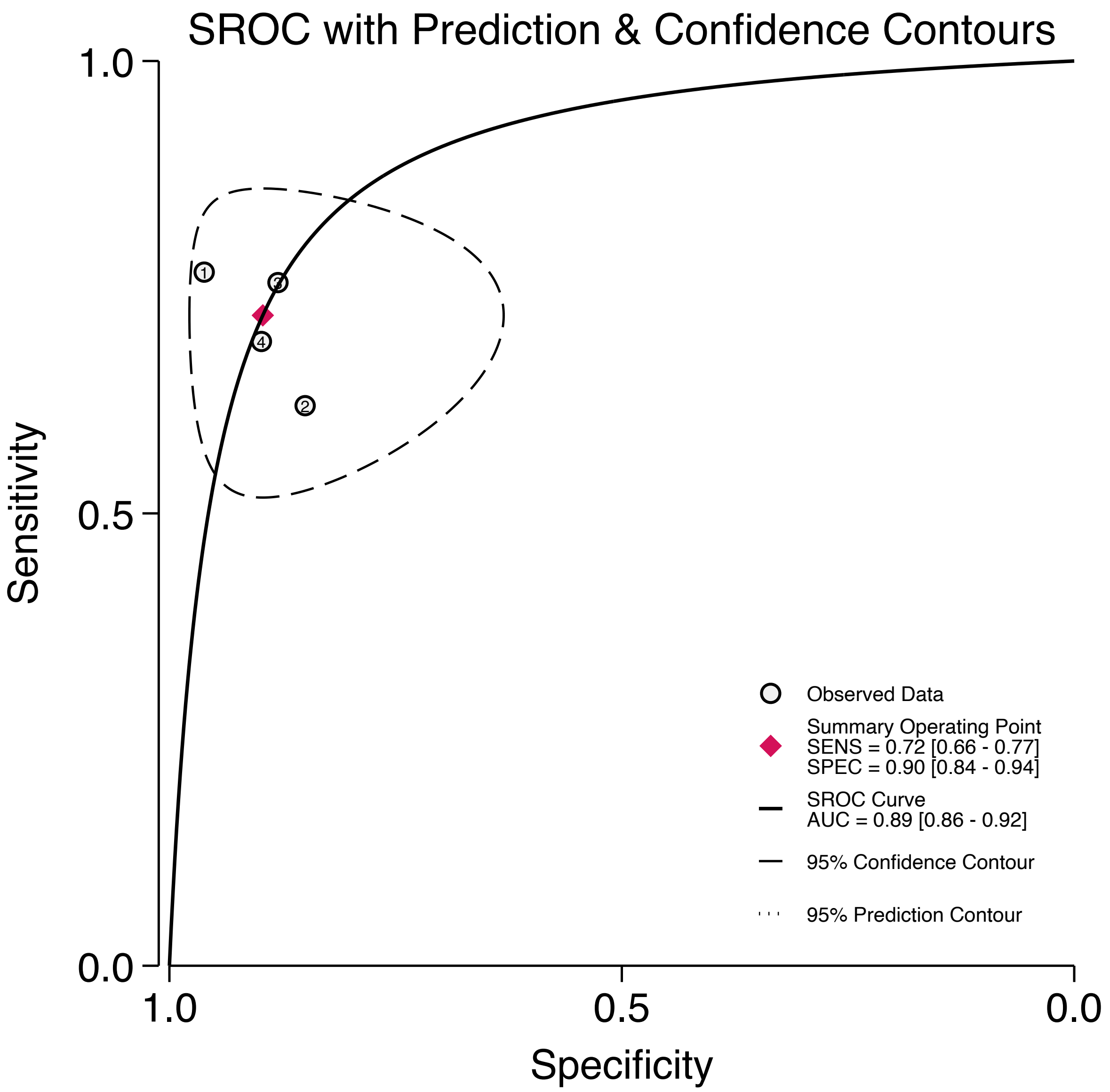

Supplementary Figure S4. Summary receiver operating characteristic (SROC) curve for miR-29a. The SROC curve illustrates the diagnostic performance of miR-29a, with an area under the curve (AUC) of 0.89 (95% CI: 0.86-0.92), indicating strong overall diagnostic performance.
